# BPS2025: A demographically focused dataset of handwritten bangla primary script for early writer recognition

**DOI:** 10.1016/j.dib.2026.112700

**Published:** 2026-03-19

**Authors:** Md Monir Ahammod Bin Atique, Md. Morshed Ali, Kashfi Shormita Kushal, Nezam Uddin, Mehedi Hasan Saurav, Md Sharifuzzaman Shajib

**Affiliations:** a Department of Computer Science and Engineering, Uttara University, Dhaka 1230, Bangladesh; b Department of Computer Science and Engineering, International University of Business Agriculture and Technology, Dhaka 1230, Bangladesh; c Department of Mathematics, Dhaka College, University of Dhaka, Dhaka 1205, Bangladesh; d Department of Management Studies, University of Dhaka, Dhaka 1000, Bangladesh; e Department of Computer Science and Engineering, Gopalganj Science and Technology University, Gopalganj 8100, Bangladesh

**Keywords:** Bangla handwriting recognition, Primary education, Dataset, Deep learning, Optical character recognition, Bengali Script, Bangladesh

## Abstract

The classification of Bangla characters and digits is a fundamental component of Natural Language Processing (NLP) and computer vision applications. However, despite advancements in handwritten character recognition (HCR) for other languages, recognizing Bangla script remains a formidable challenge, primarily due to its extensive character set, intricate compound forms, and significant stylistic variations. While existing datasets have significantly advanced the field of Bangla HCR, they frequently overlook the complexity and variability of primary-level learners' handwriting. This paper introduces the first extensive Bangla Primary Script 2025 (BPS2025) dataset, a novel, balanced and comprehensive, demographically oriented collection of isolated characters and numerals specially focused young primary school students. The dataset was selectively curated from 500 students, aged 7 to 12 and in grades 2 to 5, across four districts in Bangladesh. It comprises 24,420 raw images across 60 balanced classes, including 50 basic characters and 10 digits, which followed five stage pre-processing pipelines to process the final dataset. The dataset addresses a significant gap in existing benchmarks, which is expected to support future research and real-world educational applications by facilitating the development of robust and accurate recognition models for Bangla script.

Specifications Table

**Table:** 

Subject	Computer Sciences, Image processing, Artificial Intelligence, Educational Technology, Digital Learning.
Specific subject area	Handwriting Recognition, OCR, Machine Learning, Deep Learning, NLP, Corpus.
Data Format	Raw data (.jpg)
Type of data	Image.
Data collection	Data were collected using standardized, pre-printed forms with designated character and digit boxes to ensure consistent placement and scale. Students wrote under natural classroom conditions with black pens, and the completed forms were digitized either with a smartphone or at 300 DPI using a flatbed scanner to preserve fine-grained handwriting details for further analysis.
Data source location	Four districts across Bangladesh, including Dhaka, Chittagong, Rajshahi, and Khulna.
Data accessibility	Repository name: Mendeley Data Data identification number: 10.17632/mt6jfkxprj.3 Direct URL to data: https://data.mendeley.com/datasets/mt6jfkxprj/3 Supplementary File S1 provides a step-by-step guide to access and use the dataset. Supplementary File S2 provides the pre-processing procedures for reproducibility.
Related research article	None.

## 1 Value of the Data

• **Demographic Specificity:** BPS2025 is the first large-scale dataset focusing exclusively on the handwriting of young Bangla learners (primary school students), capturing early-stage variations that are crucial for educational OCR tools [1] and various areas of NLP, where the varied writing styles of young learners could pose significant challenges for automatic handwritten character and digit recognition, such as in the image classification and image recognition problems within the DL paradigm.
• **Balanced and Comprehensive:** The dataset includes all 50 basic Bangla characters and 10 numerals, with 407 samples per class, ensuring balanced representation for robust model training.
• **Structured for Machine Learning:** The raw images were pre-processed, labeled, and organized into a directory hierarchy compatible with DL frameworks [2], reducing pre-processing overhead. Additionally, the raw images were preserved for reference purposes.
• **Demand for Bengali Region Educational Applications:** Since many Bangladeshi students learn in Bangla [3], there is a rising need for automated educational systems in that language. BPS2025 can enable research in automated grading, writer identification, literacy tools, and adaptive learning systems tailored to early handwriting acquisition.
• **Benchmarking Potential:** This dataset provides a standardized benchmark for evaluating recognition models on children’s handwriting [4], a domain often underrepresented in existing resources. It can also be used to explore potential patterns related to age and gender in handwriting samples collected at an early stage.

## 2 Background

**Fig. 1:**
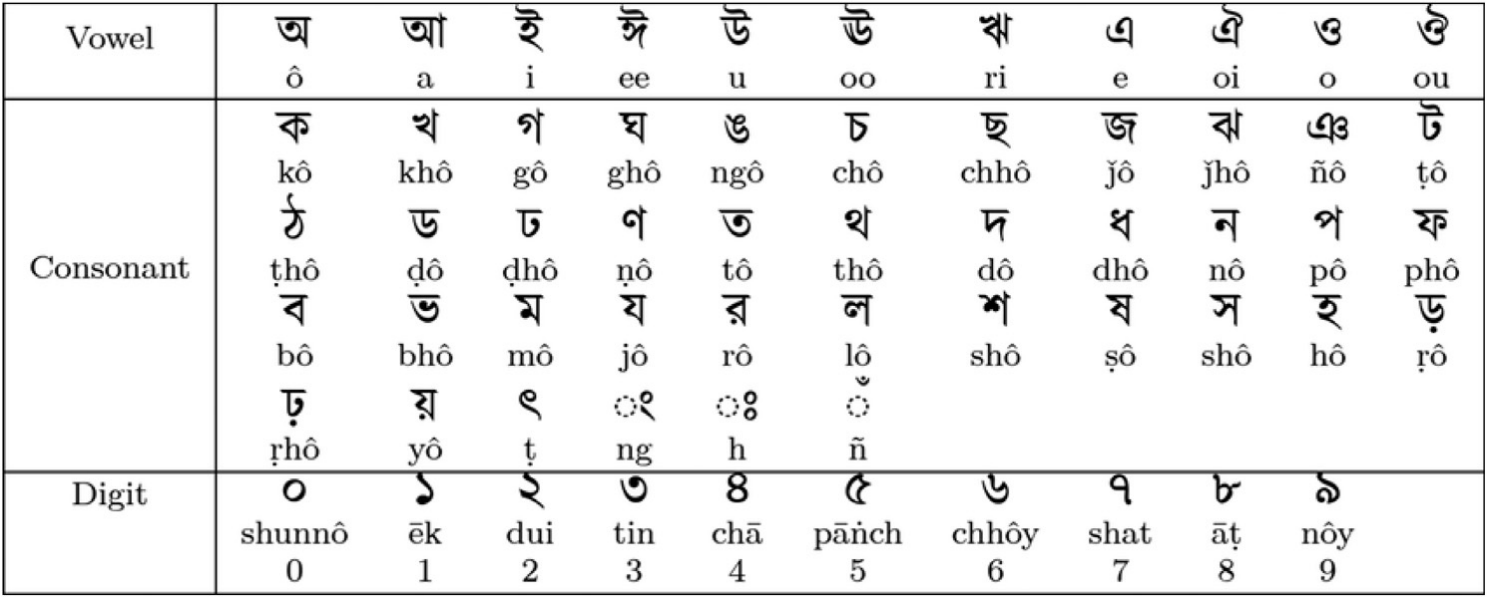
Bengali Letters (vowels and consonants) and Digits with Pronunciations [20].

HCR, an essential subfield of OCR, is a critical technology with broad applications in sectors such as healthcare, banking, finance, education, and e-learning, across many languages. It also represents one of the most challenging branches of computer vision and artificial intelligence (AI) [5]. Furthermore, it also plays a fundamental role in key processes including document digitization, automated data entry, the conversion of handwritten notes from tablets into digital text, and even specialized tasks like gender identification and deciphering doctors' prescriptions [6]. Bangla is the seventh most commonly spoken language in the world, used by >250 million people [7]. It poses special challenges to handwriting recognition due to its large character repertoire, conjunctive forms, and diacritical marks. The Bangla writing system is primarily composed of a 50-character alphabet (11 vowels and 39 consonants) and 10 distinct digits, as illustrated in Fig. 1. For the vowel and consonant sets, the corresponding English pronunciations are provided beneath each glyph to aid interpretation. The ten Bangla digits are also presented, accompanied by their numerical values and English equivalents. Numerous studies have been conducted to recognize various forms of Bangla handwriting, encompassing primary characters, compound characters, special symbols, and numerals. The latest developments in DL have made recognition more accurate; however, most available datasets, including BanglaLekha-Isolated [8] and CBD2023 [9], are based on adult or general population handwriting. Bangla REX [10] presents a substantial dataset specifically tailored for relation extraction tasks in the Bangla language. NOIRBETTIK [11] is developed for completing multiple-choice questions based on reading comprehension and BanglaTense [12] is designed for categorizing tense in Bangla. Some other dataset focused on Bangla sentences that are helpful for sentiment analysis in Bengali region. BanglaMUSE [13] contains a manually created sentiment-annotated Bangla text and corresponding speech recordings. BanglaBlend [14] is developed with 7350 annotated sentences categorized by saint and common form of Bangla language. BANGLA_ABSA [15] is designed for sentiment analysis containing information and comments about car, mobile phone, movie and restaurant. ChatgaiyyaAlap [16] and ONUBAD [17] are curated with a view to recognizing Bangla dialects. Shomikoron [18] is specially created for mathematical text and equation in Bangla. KBES [19] presents Bangla speech emotion with their intensity level. While these datasets are useful, they are not representative of young learners' handwriting, which is more variable and exhibits formative errors and developmental characteristics typical of an early learning stage.

In this research, the BPS2025 dataset fills this gap through a systematic sampling of handwriting from primary school students across multiple cities in Bangladesh. By incorporating the natural writing of children, BPS2025 facilitates the development of recognition systems tailored to AI educational contexts. These include automated evaluation systems and online literacy platforms, specifically targeting the vast number of primary and elementary schools in Bangladesh where teaching resources are limited.

## 3 Data Description

**Fig. 2:**
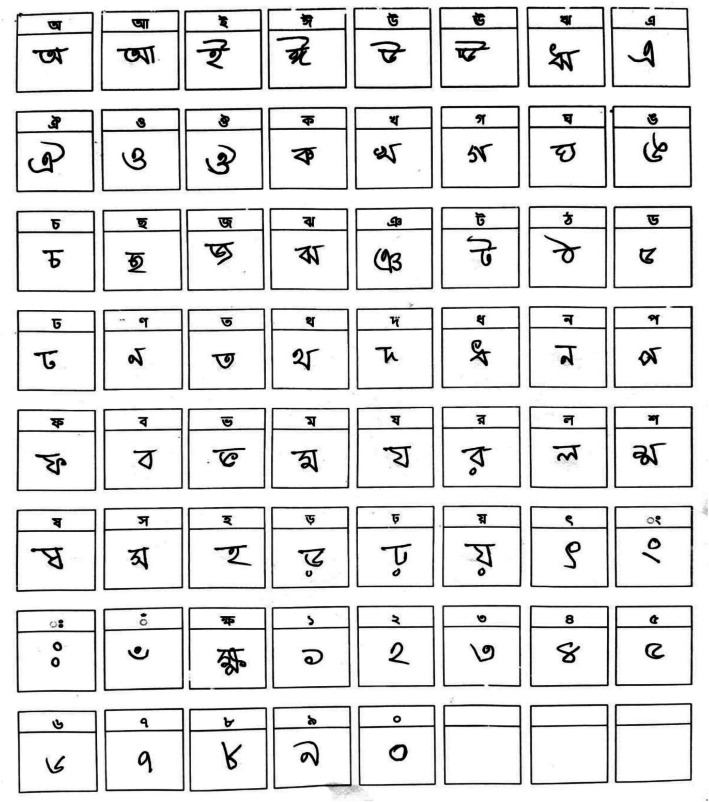
A Sample Raw Completed Scripts of Handwritten Bangla Alphabets and Digits from BPS2025 Dataset.

The BPS2025 dataset consists of 24,420 processed images of isolated Bangla characters and digits, collected from 500 students aged 7–12 across schools in four districts of Bangladesh. The dataset images represent 50 letters (11 vowels and 39 consonants) and 10 numerals, resulting in 60 total classes. After removing incomplete, scribbled, or physically damaged samples, 407 samples remained per class, resulting in a balanced dataset suitable for supervised learning. Along with the cropped raw images, the original full-page scans (before cropping) are also provided, as they are often valuable for researchers testing automated grid detection and layout analysis algorithms. The link of the images has been attached in the Supplementary File S1. Table 1 provides an overview of the collected BPS2025 dataset, including the component type, number of classes, samples per class, and total samples for each component. To illustrate the data collection strategy of the proposed BPS2025 dataset, Fig. 2 presents a sample raw snapshot of a completed script form.

**Table 1:** BPS2025 Dataset Summary.

Component	No. of Classes	Samples per Class	Total Samples
Digits	10	407	4070
Vowels	11	407	4477
Consonants	39	407	15,873
Total	**60**	**407**	**24,420**

**Fig. 3:**
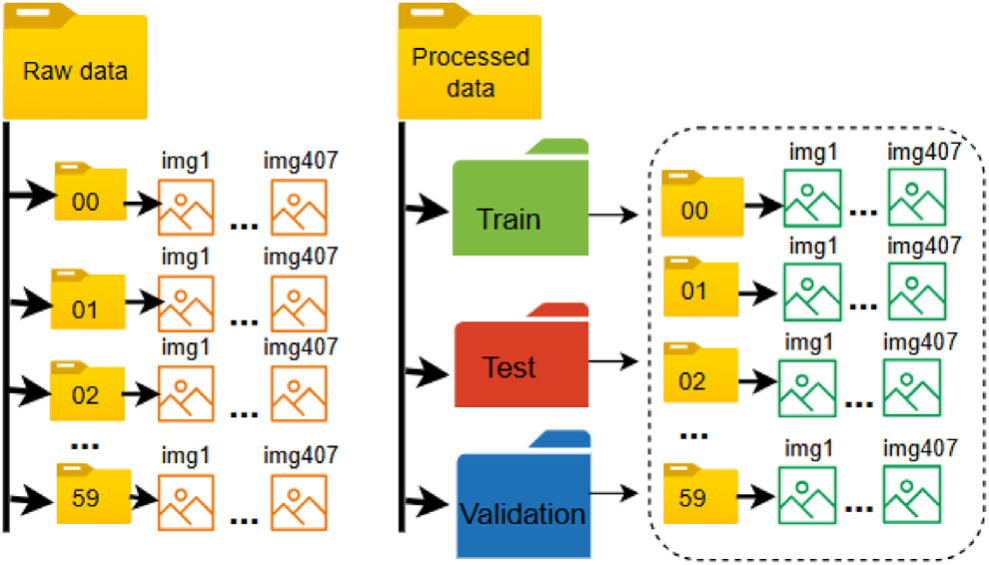
The Directory Structure of the BPS2025 Dataset.

The dataset is provided in two versions: i) the raw, unprocessed scanned data, and ii) The processed data, to which a five-stage preprocessing pipeline was applied, were resized to 120 × 120 pixels (final pixel dimensions). Each version is organized into 60 folders, named according to the class labels (00 to 59). Each folder contains all corresponding images for its class. The labels correspond to the following mapping: 00–49 represent the basic Bangla characters (vowels and consonants), and 50–59 represent the digits (0–9) which is clearly depicted in Fig 3.

## 4 Experimental Design, Materials and Methods

**Fig. 4:**
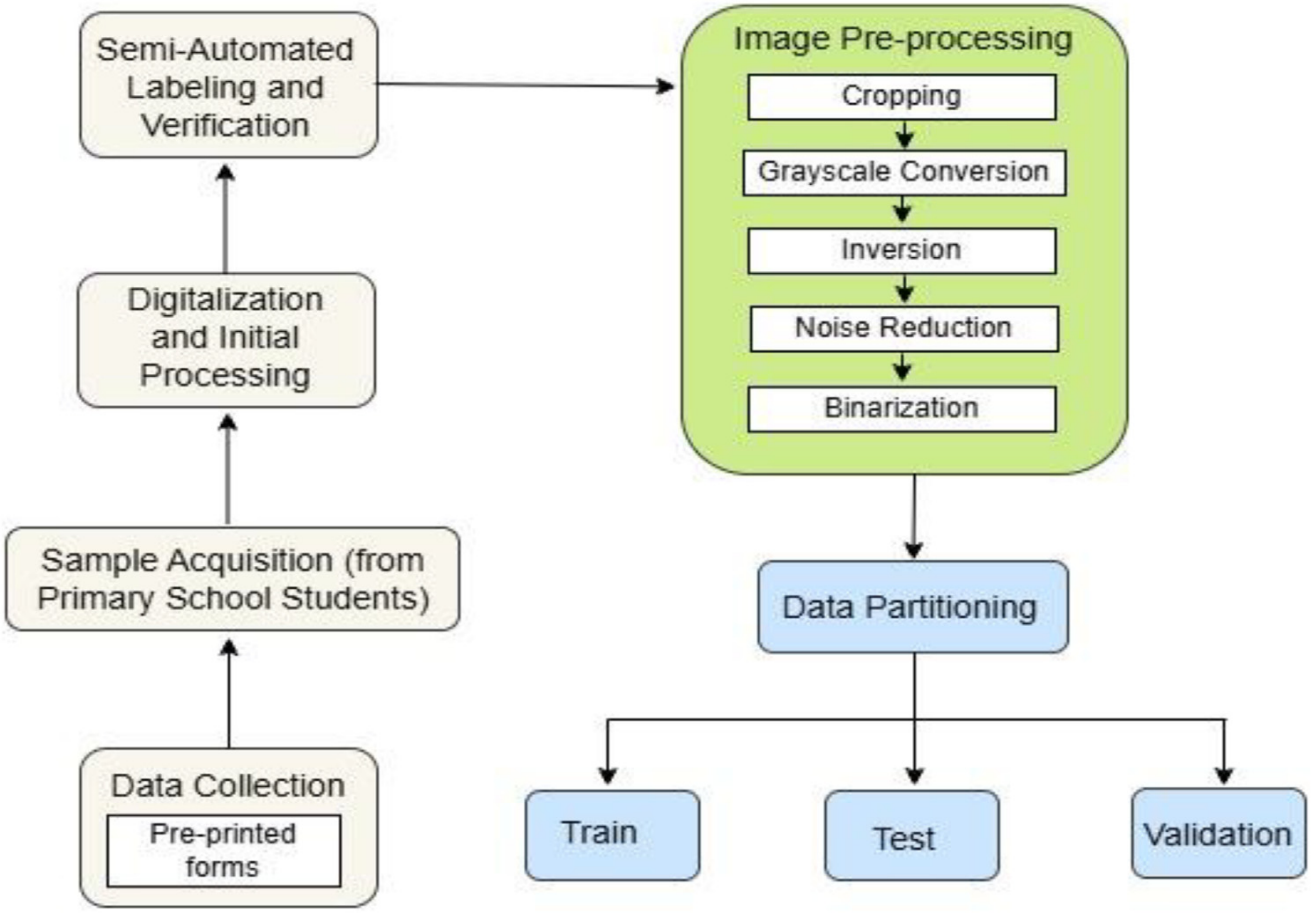
The development process of the entire BPS2025 Dataset.

In this section, Fig. 4 presents the overall procedure of making our BPS2025 dataset, including data collection with preprint forms, digitization and initial processing, labeling, image processing with necessary techniques, and finally data splitting into training, testing, and validation. Details are described in the following subsection.

### 4.1 Dataset creation motivation and participant demographics and recruitment

While there has been extensive work and impressive success in English handwritten character and digit recognition, Bangla remains far behind in matching this rapid growth, despite being the seventh most used language. However, almost all existing benchmark Bangla datasets have focused mainly on adults and the general population. We aim to focus on young learners at the elementary school level to capture various handwriting styles at an early age to make a diverse dataset.

**Fig. 5:**
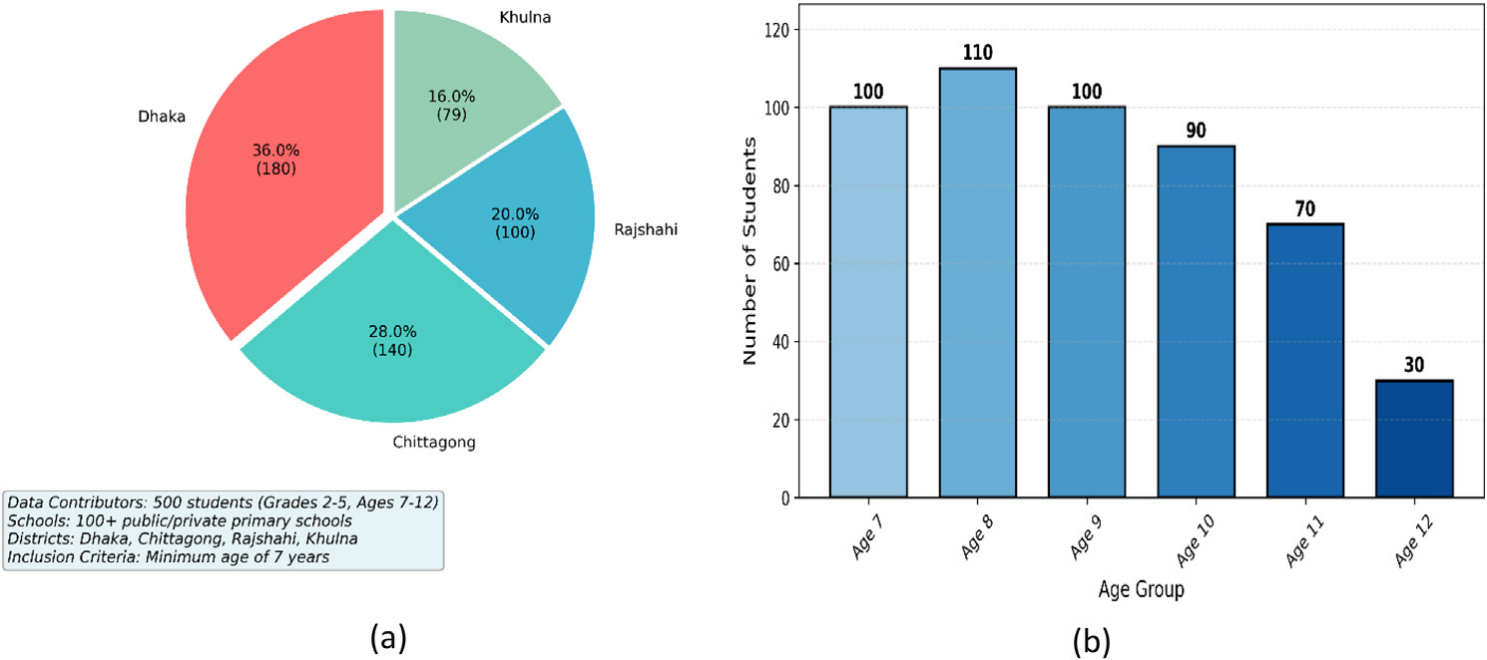
Participant geographic and age distribution analysis for a total of 500 students aged 7 to 12: (a) District-wise student distribution, and (b) Age-wise student distribution.

**Fig. 6:**
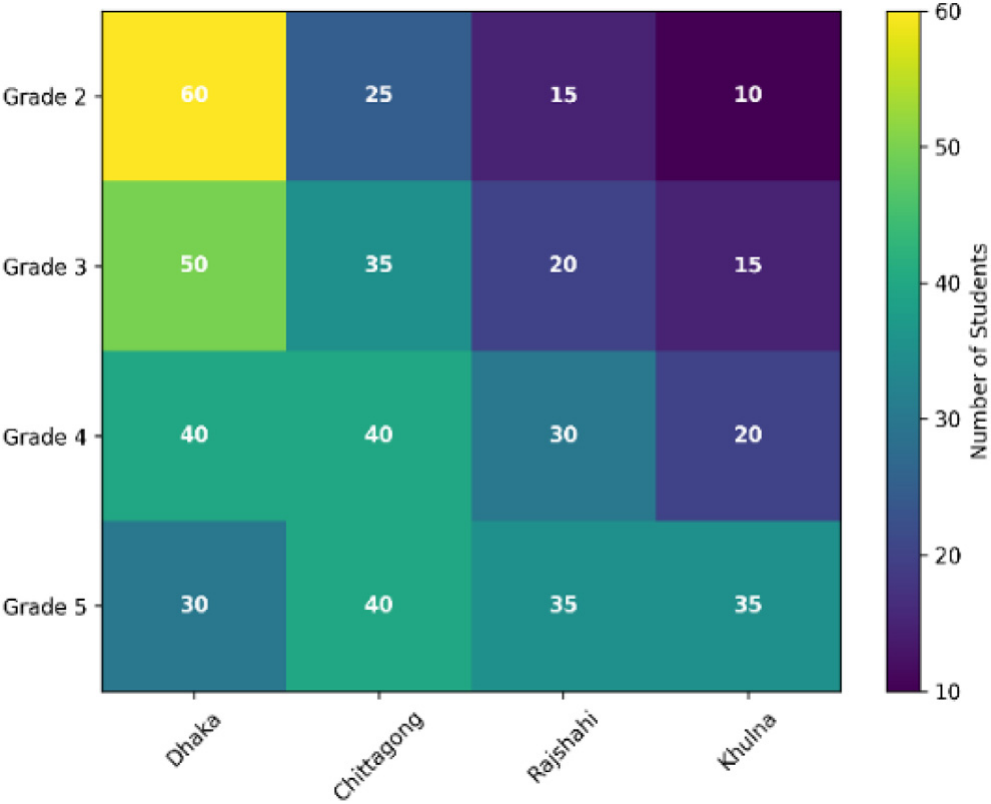
Geographic distribution of participants across four districts of Bangladesh and grade-level analysis for a total of 500 students.

In this work, the data contributors were 500 students enrolled in grades 2 through 5 across a network of over 100 public and private primary schools in four geographically diverse districts (or cities) of Bangladesh (e.g., Dhaka, Chittagong, Rajshahi, Khulna). Participants were selected based on three primary inclusion criteria: a) A minimum age of 7 years, b) Permanent residency in the district, and c) Physical fitness to complete the survey. The gender of the subjects was also recorded, with 60 % male and 40 % female participants. Participant location, age, and grade distribution are illustrated in Fig. 5. The data show that most participants were from Dhaka city, the majority of students were aged 7 to 9, and the grade distribution across the four districts is presented in Fig. 6. This age and gender distribution, combined with the multi-district sampling strategy, was designed to capture a wide range of pedagogical influences and socio-cultural backgrounds, thereby enhancing the dataset's representativeness. Informed consent was obtained from school authorities and parents prior to data collection. No personally identifiable information (PII) is retained in the publicly released dataset; all samples are anonymized.

### 4.2 Data collection protocol

A front page containing general information for the data collection form—including age, sex, grade, district, institution, and form ID (ranging from BPS_F_001 to BPS_F_500), was used. The collection was designed to simulate natural writing conditions while ensuring consistency for subsequent processing. We designed a standardized, pre-printed A4-sized form containing an 8 × 8 grid of clearly demarcated, numbered bounding boxes (2.5 cm x 2.5 cm each). A blank sample of the A4 data collection form was included in Supplementary File S1, enabling other researchers to use the identical layout for cross-dataset compatibility. Additionally, Supplementary File S1 provides a step-by-step guide to access, download, and use the dataset (raw and processed versions), including directory structure and example loading code. A total of 64 boxes (8 × 8 grid) were printed on each form, of which 60 were used for the 50 Bangla characters and 10 digits, leaving 4 boxes empty. Within each box, the target character or digit was printed in a light gray, standard font as a guide as depicted in Fig. 2. Participants were provided with the empty form with a front page and a black regular ball-point or gel pen. They were instructed to write the specified character once inside each corresponding box, following the printed guide, at their own pace in a classroom setting. This method controlled for scale and positioning, which greatly facilitated the subsequent automated cropping stage. Although 500 students participated, 93 forms were excluded due to incompleteness, scribbles, or physical damage. Consequently, 407 completed forms were collected, each containing one sample of all 60 characters.

### 4.3 Digitalization and initial processing

All 407 paper forms were digitized using two methods: initially with smartphone cameras (Samsung Galaxy S22 Ultra 5 G) under natural conditions, and subsequently for better resolution with a high-quality flatbed scanner (Epson Perfection V39) at 300 DPI, producing RGB images. This resolution was chosen to preserve fine-grained stroke details essential for character recognition while maintaining manageable file sizes. The scanned images were then deskewed automatically using a standard image processing library (OpenCV) to correct minor rotational artifacts introduced during scanning.

### 4.4 Semi-automated labeling and verification pipeline

**Fig. 7:**
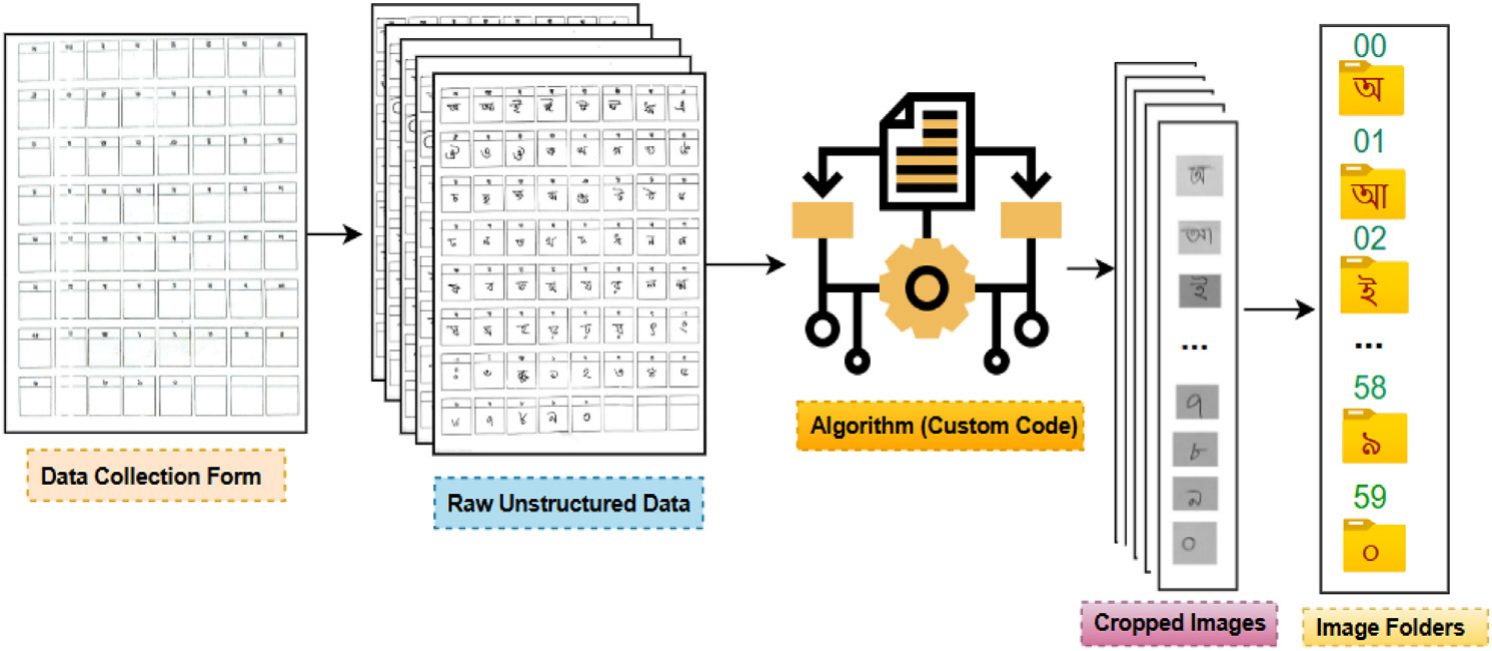
Raw Image cropping and labeling.

Labeling leveraged the structured nature of the collection form. A custom Python script using OpenCV was developed to perform the following steps: 1) Detect the outer border of the form grid; 2) Based on the known grid dimensions, calculate the pixel coordinates for each of the 60 bounding boxes; 3) Extract and save the sub-image defined by each bounding box. The script automatically assigned a provisional label (00 to 59) to each cropped image based on its sequential position on the form which was illustrated in Fig. 7. This fully automated batch process was followed by a rigorous two-stage manual verification: First, a visual check of a sample from each batch ensured the grid detection was accurate. Second, a random 10 % of all cropped images were inspected for correct content and label assignment. Any forms with significant damage or misalignment were reprocessed manually. This hybrid approach ensured high labeling accuracy with efficient scalability.

### 4.5 Image pre-processing pipeline

**Fig. 8:**
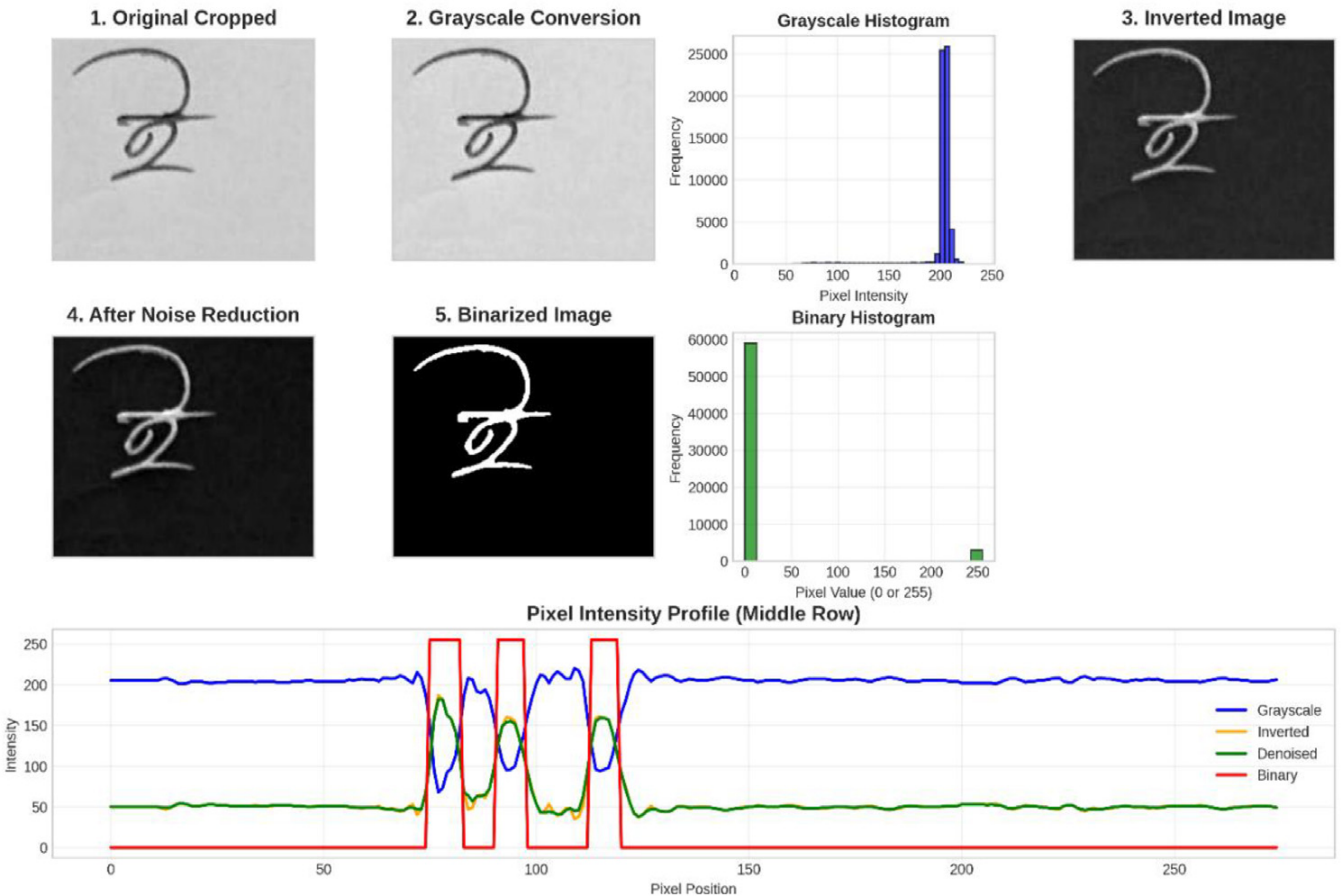
Detailed analysis of the pre-processing steps: (1) cropped raw image; (2) grayscale conversion with its grayscale histogram showing pixel-intensity frequency; (3) inversion; (4) noise reduction using a 3×3 filter; and (5) binarization with its binary histogram; the middle-row pixel-intensity profile compares grayscale (blue), inverted (orange), denoised (green), and binary (red) intensities across pixel positions, highlighting transition regions where foreground and background separate.

**Fig. 9:**
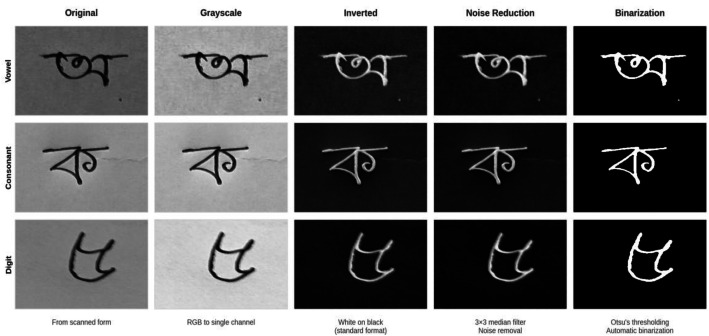
Visual illustration of the pre-processing pipeline for Bangla handwritten characters (vowel, consonant, digit): original scanned image, followed by grayscale conversion, then inversion, followed by noise reduction using a 3×3 median filter, and the final binarization using Otsu’s thresholding.

Each cropped character image underwent a standardized 5 stages pre-processing chain to normalize the data for model input, as illustrated in Fig. 8. No data augmentation was applied in this experiment to maintain the integrity and authenticity of the original dataset. The following section outlines the pre-processing steps employed in this experiment. In Fig. 9, a detailed depiction of the pre-processing workflow applied to all Bangla handwritten vowels, consonants, and digits in this work is presented. All processing was performed in Python using OpenCV (cv2) for image I/O and transformations (resizing, grayscale conversion, inversion, median filtering, and Otsu thresholding), with NumPy and standard Python libraries supporting array operations and file management. For full reproducibility, the pre-processing code is provided as Supplementary File S2, including the original script used during dataset preparation and a non-interactive command-line interface (CLI) runner.

• **Cropped Image:** After data cropping and labeling, all cropped images are organized into 60 sequential folders (00 → ‘’ (a in English, short “o”), 59 → ‘’ (0 in English)).
• **Grayscale Conversion:** All cropped RGB images were then converted to a single-channel grayscale format using the luminance formula *Y* = 0.299R + 0.587 *G* + 0.114B
• **Inversion:** The image was then inverted (*I* = 255 - I) to produce a white character stroke on a uniform black background, a standard convention in document image analysis.
• **Noise Reduction:** Following inversion, a 3 × 3 median filter was applied to reduce salt-and-pepper noise and minor scanner artifacts, as this kernel size effectively suppresses isolated noise while preserving fine edge and stroke details in this work.
• **Binarization:** Finally, the Otsu's global thresholding algorithm was applied to the filtered grayscale image to automatically determine an optimal threshold value, converting the image into a binary map (pixel values 0 or 255).

### 4.6 Data partitioning

**Fig. 10:**
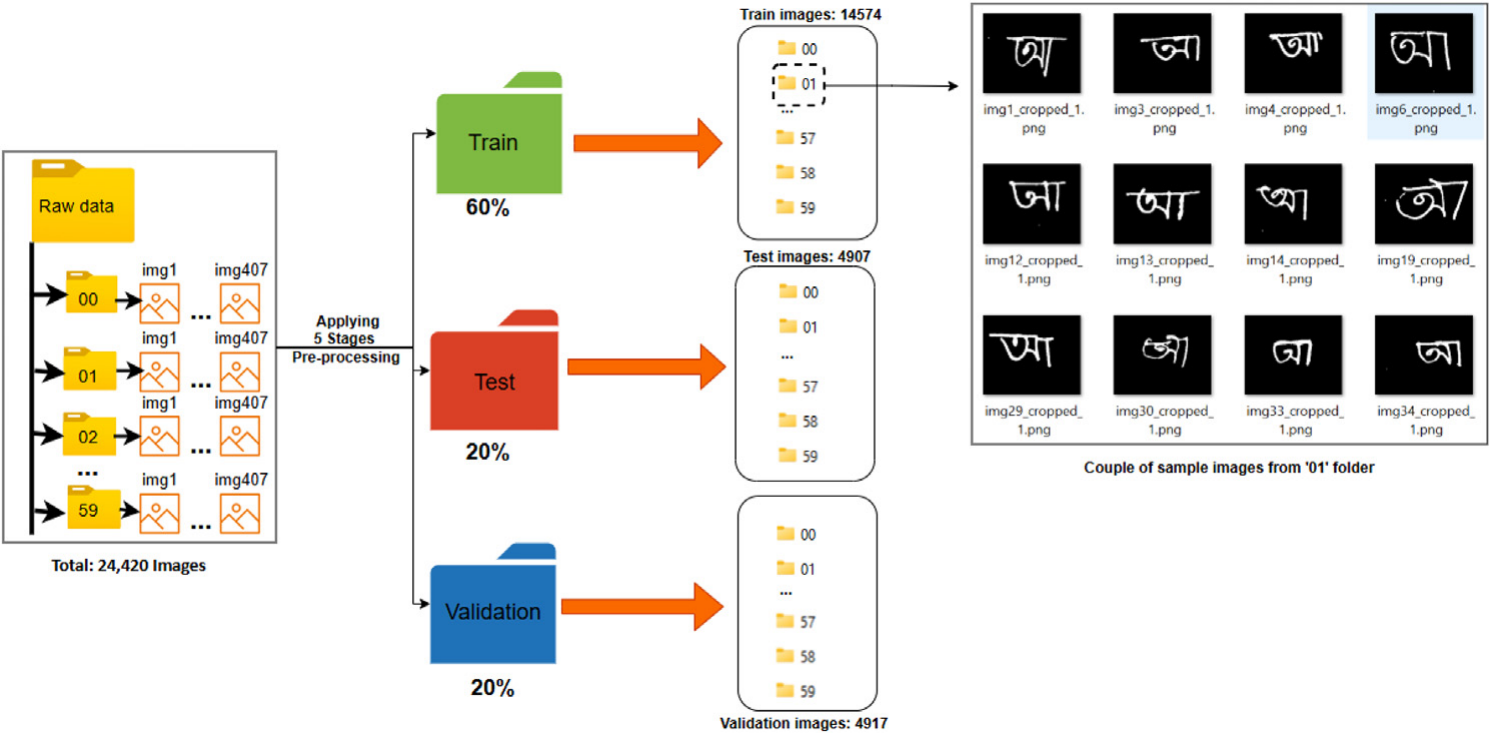
Dataset partitioning strategy after applying the five-stage pre-processing pipeline.

Following pre-processing, the fully processed dataset of 24,420 images was randomly shuffled at the class level to ensure a uniform distribution of writing styles across splits. It was then partitioned into training (60 %, 14,652 images), validation (20 %, 4884 images), and test (20 %, 4884 images) sets, which was visually depicted in Fig. 10. This partition is fixed and provided in the repository to ensure consistent benchmarking across future studies.

## Data Accessibility

BPS2025: A Demographically Focused Dataset of Handwritten Bangla Primary Script for Early Writer Recognition (Raw and Processed Data) (Mendeley Data)

BPS2025 (Github Link)

## Limitations

The BPS2025 dataset is intentionally scoped to isolated, basic characters and numerals. It does not include compound characters (juktakkhors) [21], diacritic modifiers (kar-fola), or continuous cursive handwriting. Therefore, models trained exclusively on BPS2025 are not directly applicable to the recognition of full Bangla text. Furthermore, the dataset is specific to the handwriting of Bangladeshi primary school students. While this is its core contribution, it may limit generalizability to the writing styles of adults, individuals from the Bengali-speaking regions of India (e.g., West Bengal), or children educated under significantly different pedagogical systems.

## Ethics Statement

The authors have read and followed the ethical requirements for publication in Data in Brief and confirm that the current work does not involve human subjects, animal experiments, or any data collected from social media platforms.

## CRediT Author Statement

**Md Monir Ahammod Bin Atique:** Conceptualization, Methodology. **Md. Morshed Ali:** Supervision, Writing, Reviewing and Editing **Kashfi Shormita Kushal:** Visualization, Original Draft Preparation. **Nezam Uddin:** Writing- Reviewing and Editing. **Mehedi Hasan Saurav:** Software, Validation. **Md Sharifuzzaman Shajib:** Data Curation.

## Data Availability

Mendeley DataBPS2025: A Demographically Focused Dataset of Handwritten Bangla Primary Script for Early Writer Recognition (Original data).

## Appendix Supplementary materials

Supplementary File S1 provides a step-by-step guide to access, download, and use the dataset (original full-page scans, raw and processed versions), including directory structure and example loading code. Supplementary File S2 provides the pre-processing procedures for reproducibility. Image, application 1
