# Supplementary material for: BPS2025: A demographically focused dataset of handwritten bangla primary script for early writer recognition
Source: Data Brief. 2026 Mar 19;66:112700. doi: 10.1016/j.dib.2026.112700 (PMC13062511; doi:10.1016/j.dib.2026.112700)
Supplement: Supplementary file 1 — Supplementary File S1 provides a step-by-step guide to access, download, and use the dataset (original full-page scans, raw and processed versions), including directory structure and example loading code. Supplementary File S2 provides the pre-processing procedures for reproducibility. [file mmc1.zip › Supplementary/Supplementary_File_S1_BPS2025_Access_and_Usage_Guide.docx]

Supplementary File S1. BPS2025 Dataset Access and Usage Guide

This guide describes how to access, download, and use the Bangla Primary Script 2025 (BPS2025) dataset (raw and processed versions), including recommended directory structure and example loading code.

# S1.1 Repository locations and citation

Primary repository (archival): Mendeley Data — DOI: 10.17632/mt6jfkxprj.3

Landing page: https://data.mendeley.com/datasets/mt6jfkxprj/3

Documentation (GitHub repository): https://github.com/monircse061/BPS2025

License: Creative Commons Attribution 4.0 (CC BY 4.0).

# S1.2 Download instructions

Option A — Mendeley Data (recommended for archival citation):

- Open the Mendeley Data landing page.
- Click “Download All” (or download files individually) and extract the ZIP archive.
- Verify that you have either (i) raw images only, or (ii) both raw and processed images, depending on your chosen download.

Option B — GitHub (recommended for quick access to documentation/scripts):

- Open the GitHub repository.
- Use the “Code → Download ZIP” option (or clone with Git).
- Note: Large datasets may be hosted primarily on Mendeley; GitHub may provide documentation/scripts and may mirror a lightweight subset.

# S1.3 Data collection form

During the data collection phase, we employed a standardized A4-sized form (see **Fig. 1**). This template ensures consistency and may facilitate cross-dataset compatibility by allowing other researchers to adopt the exact same layout.


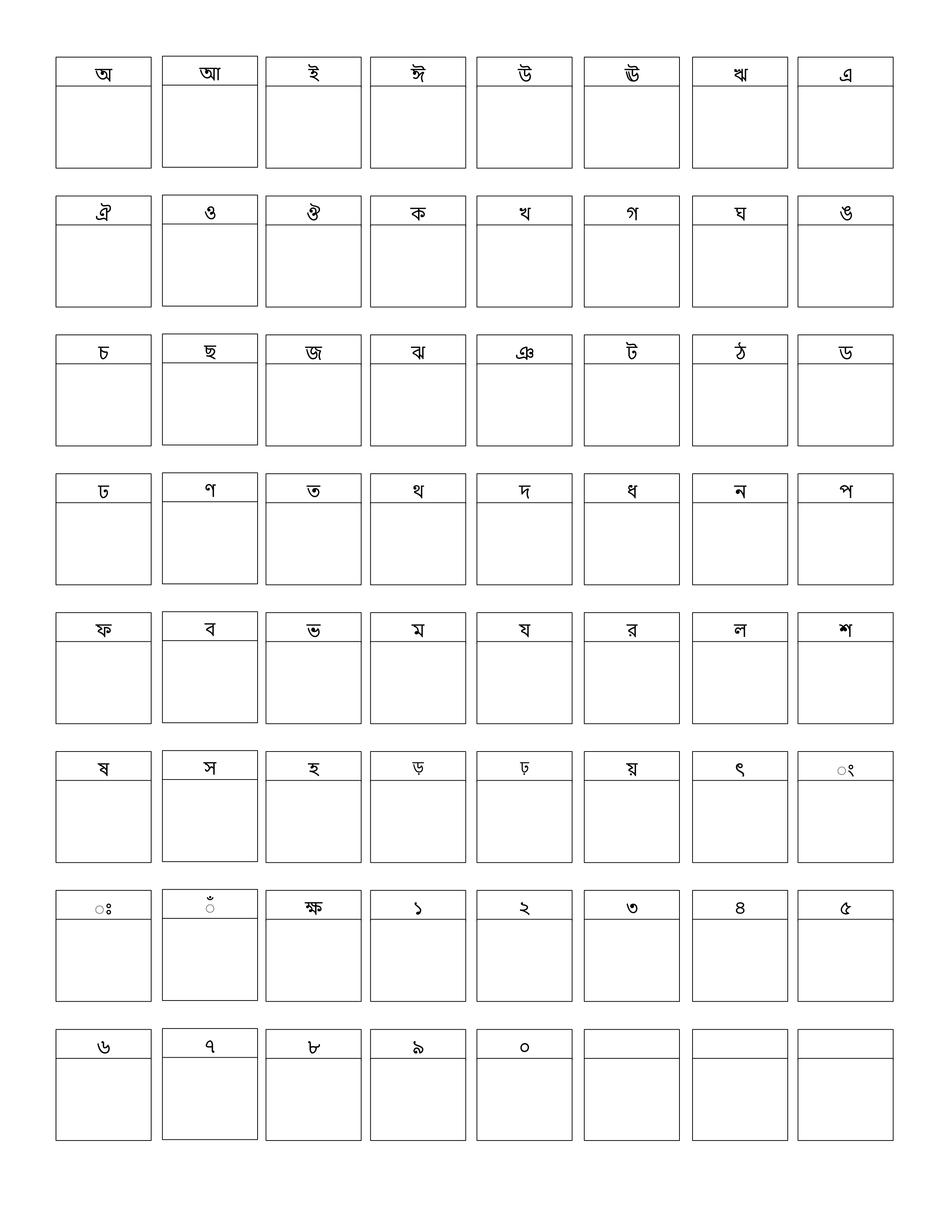


**Figure 1.** Data Collection Form (Blank Template).

# S1.4 Data Collection Front Page

# A front page containing general information for the data collection form—including age, sex, grade, district, institution, and form ID (ranging from BPS_F_001 to BPS_F_500)—was used, as depicted in below in Fig. 2.

**
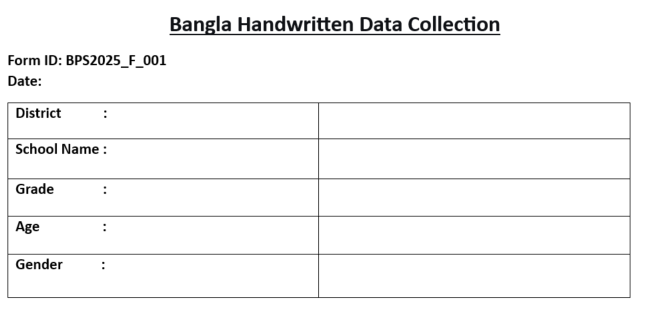
**

**Figure 2.** Data Collection Front Page

# S1.5 Dataset contents and organization

BPS2025 contains 24,420 isolated handwritten images across 60 balanced classes (50 Bangla basic characters and 10 digits).

The dataset is offered in **two versions**: (i) raw scanned/cropped images and (ii) processed images produced by a standardized preprocessing pipeline.

Preprocessing scripts are provided in Supplementary File S2 (binary_code.py: original script used during dataset preparation; bps2025_preprocess_cli.py: non-interactive CLI runner for easy reproduction).

Label mapping and folder naming:

- Each class is stored in a two-digit folder name from 00 to 59.
- Folders 00–49 correspond to Bangla basic characters (11 vowels and 39 consonants).
- Folders 50–59 correspond to Bangla digits (0–9).

Typical directory structure (processed version):

BPS2025/
 train/
 00/ 01/ ... 59/
 validation/
 00/ 01/ ... 59/
 test/
 00/ 01/ ... 59/

# S1.6 Quickstart: loading the dataset in Python

Example (framework-agnostic): iterate through folders and collect (path, label) pairs.

from pathlib import Path

root = Path("BPS2025") # change this to your extracted dataset path
split = "train" # "train", "validation", or "test"
split_dir = root / split

samples = []
for class_id in range(60):
 cls = f"{class_id:02d}"
 for img_path in (split_dir / cls).glob("*.*"):
 samples.append((img_path, class_id))

print("num samples:", len(samples))
print("first sample:", samples[0])

Example (PyTorch-style Dataset skeleton):

import cv2
import torch
from torch.utils.data import Dataset
from pathlib import Path

class BPS2025Dataset(Dataset):
 def __init__(self, root, split="train", transform=None):
 self.root = Path(root) / split
 self.transform = transform
 self.items = []
 for y in range(60):
 cls = f"{y:02d}"
 for p in (self.root / cls).glob("*.*"):
 self.items.append((p, y))

 def __len__(self):
 return len(self.items)

 def __getitem__(self, idx):
 path, y = self.items[idx]
 img = cv2.imread(str(path), cv2.IMREAD_GRAYSCALE)
 if img is None:
 raise RuntimeError(f"Failed to read: {path}")
 if self.transform:
 img = self.transform(img)
 else:
 img = torch.from_numpy(img).unsqueeze(0).float() / 255.0 # (1,H,W)
 return img, y

# S1.7 Notes for reproducible experiments

- If you use the processed version, keep the provided train/validation/test split unchanged for benchmark comparability.
- If you use the raw version, apply the preprocessing script in Supplementary File S2 to reproduce the processed images.
- Report whether your model uses raw grayscale, inverted grayscale, or binarized images as input, and specify the final image resolution used by your training pipeline.

# S1.8 Original full-page scans images

Along with the cropped raw images, the original full-page scans (before cropping) are also provided, as they are often valuable for researchers testing automated grid detection and layout analysis algorithms.

Original full-page scan images link: <https://drive.google.com/drive/folders/1js8VN9mrAC-owwkyWC4-Yrba5HkvTPma?usp=sharing>
