# Supplementary material for: BPS2025: A demographically focused dataset of handwritten bangla primary script for early writer recognition
Source: Data Brief. 2026 Mar 19;66:112700. doi: 10.1016/j.dib.2026.112700 (PMC13062511; doi:10.1016/j.dib.2026.112700)
Supplement: Supplementary file 1 — Supplementary File S1 provides a step-by-step guide to access, download, and use the dataset (original full-page scans, raw and processed versions), including directory structure and example loading code. Supplementary File S2 provides the pre-processing procedures for reproducibility. [file mmc1.zip › Supplementary/Supplementary_File_S2_BPS2025_Preprocessing_Scripts_and_Procedures_v2.docx]

Supplementary File S2. BPS2025 Preprocessing Scripts and Detailed Procedures

This supplementary file provides (i) the preprocessing procedure applied to the dataset and (ii) the corresponding Python script(s) used to generate the processed BPS2025 images, to support full reproducibility.

# S2.1 Summary of the preprocessing pipeline

The manuscript describes a standardized preprocessing chain applied to each cropped character image (grayscale conversion, inversion, median filtering, and Otsu binarization). In addition, our batch script optionally normalizes image size prior to processing.

- Input: cropped RGB image (per class folder 00–59).
- Optional size normalization: resize to 120×120 pixels (configurable).
- Grayscale conversion: convert to 1-channel image.
- Inversion: I := 255 − I (white strokes on black background).
- Noise reduction: 3×3 median filter.
- Binarization: Otsu global thresholding (output pixels ∈ {0,255}).

# S2.2 Provided script and how to run it

Included scripts: (i) binary_code.py (original script used during dataset preparation); (ii) bps2025_preprocess_cli.py (non-interactive CLI runner implementing the same pipeline and parameters for easy reproduction).

Dependencies:

- Python 3.8+
- opencv-python (cv2)
- numpy
- tqdm
- matplotlib (only used for sample visualization)

Expected input directory structure (processed-split input):

<base_dir>/
 train/00..59/*.jpg|png|...
 validation/00..59/*.jpg|png|...
 test/00..59/*.jpg|png|...

Outputs (created under <base_dir>/processed_bps/):

processed_bps/
 train/00..59/*.png
 validation/00..59/*.png
 test/00..59/*.png
 processed_samples.png
 processing_report.txt

Running the script (interactive):

- Open a terminal in the directory containing binary_code.py.
- Run: python **binary_code.py**
- When prompted, enter the dataset base directory path.
- Optionally enter a target size (default 120 120).
- Confirm processing with 'y'.

# S2.3 Pseudocode

for split in {train, validation, test}:
 for class_id in {00..59}:
 for image in split/class_id:
 img = read(image)
 img = resize(img, 120x120)
 gray = to_grayscale(img)
 inv = 255 - gray
 den = median_filter(inv, k=3)
 bin = otsu_threshold(den)
 save(bin, processed_bps/split/class_id)

# S2.4 Non-interactive CLI version

For convenience in automated pipelines, we also provide a non-interactive CLI wrapper (**bps2025_preprocess_cli.py**) that performs the same steps with command-line arguments (input_dir, output_dir, target_size).
